# Hyperexcitability in young iPSC-derived *C9ORF72* mutant motor neurons is associated with increased intracellular calcium release

**DOI:** 10.1038/s41598-022-09751-3

**Published:** 2022-05-05

**Authors:** Sarah Burley, Dayne A. Beccano-Kelly, Kevin Talbot, Oscar Cordero Llana, Richard Wade-Martins

**Affiliations:** 1 Department of Physiology, Anatomy and Genetics, University of Oxford, South Parks Road, Oxford, UK; 2 Nuffield Department of Clinical Neurosciences, University of Oxford, John Radcliffe Hospital, Oxford, UK; 3 Present Address: School of Biology, University of St Andrews, North Haugh, St Andrews, UK; 4 Present Address: UK Dementia Research Institute, University of Cardiff, Hadyn Ellis Building, Maindy Road, Cardiff, CF24 4HQ UK; 5 Present Address: Bristol Medical School, Translational Health Sciences, University of Bristol, Dorothy Hodgkin Building, Whitson Street, Bristol, BS1 3NY UK

**Keywords:** Amyotrophic lateral sclerosis, Induced pluripotent stem cells, Patch clamp

## Abstract

A large hexanucleotide repeat expansion in the *C9ORF72* gene is the most prevalent cause of amyotrophic lateral sclerosis (ALS). To better understand neuronal dysfunction during ALS progression, we studied motor neuron (MN) cultures derived from iPSC lines generated from *C9ORF72 (C9)* expansion carriers and unaffected controls. *C9* and control MN cultures showed comparable mRNA levels for MN markers *SMI-32, HB9* and *ISL1* and similar MN yields (> 50% TUJ1/SMI-32 double-positive MNs). Using whole-cell patch clamp we showed that *C9*-MNs have normal membrane capacitance, resistance and resting potential. However, immature (day 40) *C9*-MNs exhibited a hyperexcitable phenotype concurrent with increased release of calcium (Ca^2+^) from internal stores, but with no changes to Na_V_ and K_V_ currents. Interestingly, this was a transient phenotype. By day 47, maturing *C9*-MNs demonstrated normal electrophysiological activity, displaying only subtle alterations on mitochondrial Ca^2+^ release. Together, these findings suggest the potential importance of a developmental component to *C9ORF72*-related ALS.

## Introduction

Amyotrophic lateral sclerosis (ALS) is the most common form of adult-onset motor neuron disease (MND) and leads to paralysis and death, typically within 2–3 years of diagnosis, with only 10–20% of patients surviving ≥ 5 years^1^. The *C9ORF72* hexanucleotide repeat expansion (G4C2) accounts for 5–7% of sporadic MND and frontotemporal dementia (FTD), and 40% and 25% of familial ALS and FTD, respectively^2,3^. ALS causes the selective death of motor neurons (MNs) in the motor cortex, brainstem and spinal cord^4^. With electrophysiological activity central to neuronal function, understanding electrophysiological dysfunction in MNs carrying the *C9ORF72* expansion (*C9*-MNs) may provide important information on disease aetiology.

Neuronal hyperexcitability is often reported in embryonic and early postnatal rodent models of *SOD1* ALS^5–8^, as well as in a high proportion of sporadic and *SOD1* ALS patients^9–11^. By contrast, variation in MN excitability caused by *C9ORF72* expansions is still an emerging and complex phenotype studied in mouse models and induced pluripotent stem cell (iPSC)-derived neurons, with a range of findings reported. *C9* mouse models are limited by their inability to fully recapitulate the disease phenotypes seen in patients^12–14^ and neuronal excitability phenotypes are therefore not well established. iPSC-derived *C9*-MNs also show varied neuronal excitability phenotypes with groups reporting either hyper- or hypo- excitability^15–18^. Wainger et al*.*^15^ reported hyperexcitable spontaneous activity in *C9*-MNs using multielectrode arrays; Sareen et al*.*^16^ showed hypoexcitability, studying evoked action potential (AP) firing over a longer time course, whereas Devlin et al*.*^17^ observed a transition from hyper- to hypo-excitable behaviour over time. Hypoexcitability was defined as a reduction in the proportion of neurons able to fire repetitive APs. The same study also reported a decrease in Na_V_ and K_V_ currents over time during the switch from the hyper- to hypo-excitability phenotype. In contrast to the above ‘mixed’ culture models which also contained glial cells, Selvaraj et al*.*^18^ used a ‘pure’ MN culture and did not observe any evoked excitability changes between *C9* lines and control lines (CTRL). The authors of that study proposed a ‘glial-mediated non-cell autonomous mechanism’ accounting for the previously observed excitability changes^18^.

Previous studies used various methods for measuring neuronal excitability and resulted in a lack of understanding of the proposed mechanisms underlying the changes in excitability. In this study, we used whole-cell patch clamp electrophysiology to evoke APs and compare the excitability of a physiologically relevant neuronal/glial mixed population of iPSC-derived *C9*-MNs at early (day 40) and late (day 47) stages of maturity. We demonstrate hyperexcitability is paralleled by increased intracellular Ca^2+^ release without changes to Na_V_ and K_V_ currents in *C9* cultures at day 40. The hyperexcitability phenotype is lost by day 47 and coincides with a normalization of intracellular Ca^2+^ release, with parallel dysfunction in mitochondrial Ca^2+^ emerging. Our work highlights a developmental component to ALS by showing an early disease-relevant disruption of neurophysiology which may impact on the pathophysiology of neurons later in life.

## Results

### Differentiation of *C9ORF72* and control iPSCs to enriched spinal MN cultures in vitro

**Figure 1:**
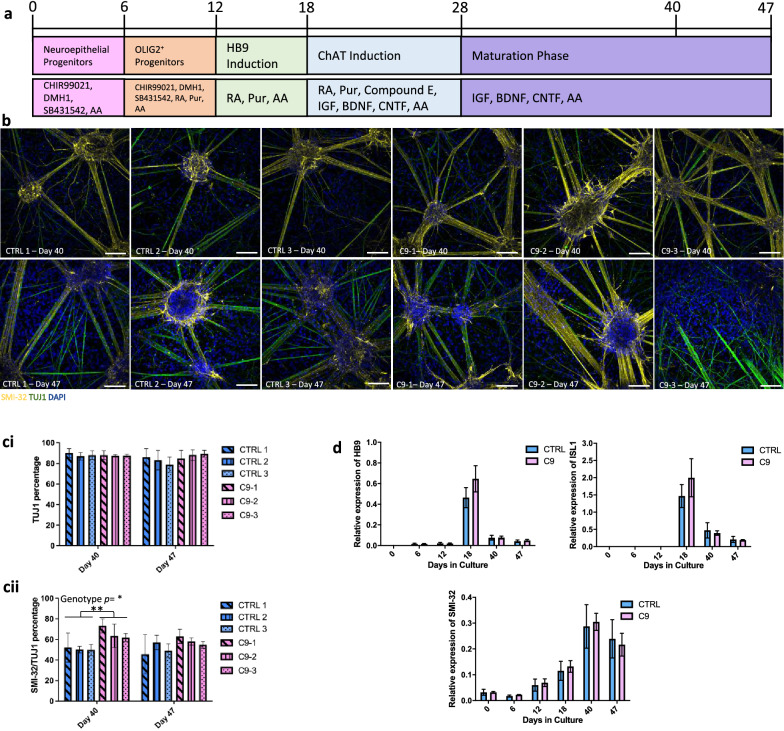
Characterization of *C9ORF72* MNs and CTRL MNs. (**a**) Diagram of the MN protocol adapted from Du et al.^20^. (**b**) iPSC-derived MNs immunostained for SMI-32 and TUJ1. Scale bars = 100 µm. (**c**) Flow cytometry of cells stained with TUJ1 and SMI-32 to verify MN counts (mean ± SEM), analysed using FlowJo v10.4 Software (BD Life Sciences). Two-way RM ANOVA: Genotype *p* = 0.0233, Time *p* = ns, Interaction *p* = ns with Sidak’s multiple comparisons, **p < 0.01, N = 3 lines, 3 differentiations (**d**) RT-qPCRs for *HB9*, *ISL1* and *SMI-32* (mean ± SEM). Two-way RM ANOVA: Genotype *p* = ns, Time *p* = < 0.0001, Interaction *p* = ns, N = 3 lines, 2 differentiations.

Three iPSC lines carrying the *C9ORF72* expansion (*C9*-1, -2, and -3) and three CTRL (CTRL-1, -2 and -3) lines were previously generated by Sendai Virus reprogramming^19^ (Figure S1) with the respective presence or absence of the G_4_C_2_ expansion confirmed using repeat primed PCR (Figure S1). A modified version of the previously published MN differentiation protocol by Du et al.^20^ was used to direct all lines towards a spinal MN lineage (Fig. 1a). Data presented in this study were obtained from multiple independent differentiations of *C9* and CTRL iPSC lines.

Using immunocytochemistry to assess differentiation efficiency, we showed an enriched percentage of double positive SMI-32/TUJ1 MNs in all lines at day 40 and 47 (Fig. 1b). Analysis by flow cytometry confirmed all lines produced > 80% TUJ1 positive neurons at day 40 and 47 (Fig. 1ci). Over 50% of neurons also co-stained for the MN marker SMI-32, with a significant increase in co-staining noted for *C9* lines vs CTRLs at day 40 (Fig. 1cii). Our protocol generates a mixed culture model enriched in MNs, with the majority of the remaining ~ 20% of non-neuronal cells represented by GFAP-positive astrocytes as measured by immunocytochemistry (Figure S1). mRNA expression of *SMI-32,* as well as transient MN markers *HB9* and *ISL1,* at day 40 and 47 showed no difference between the genotypes or time points (Fig. 1d).

### *C9ORF72* iPSC-derived MNs show early hyperexcitability

**Figure 2:**
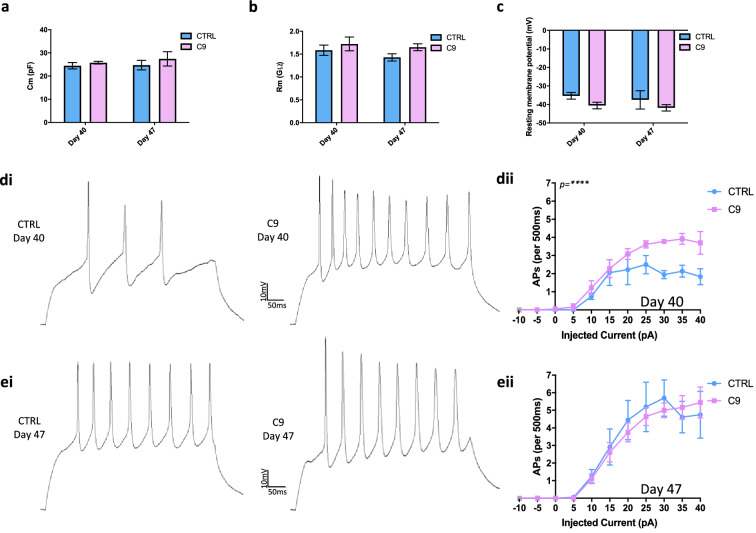
Hyperexcitability is present in young *C9ORF72* MNs vs CTRL MNs. Whole-cell patch clamp of iPSC-derived MNs showed that (**a**) membrane capacitance (*C*_m_), (**b**) membrane resistance (*R*_m_), and (**c**) resting membrane voltage were not significantly different between *C9* and CTRL MNs at day 40 nor at day 47. Two-Way RM ANOVA: *p* = ns, N = 3 lines, 1–2 differentiations. (**di**,**ei**) Representative traces of evoked action potential firing. (**dii**,**eii**) Average action potential firing per 500 ms for each current step (mean ± SEM). Non-linear regression ****p = < 0.0001.

Whole-cell patch-clamp recordings at day 40 and 47 revealed no significant difference in passive properties between *C9* or CTRL MNs (Fig. 2a–c). However, at day 40 evoked AP firing frequency was significantly higher in *C9* neurons vs CTRL neurons (Fig. 2di,ii, Figure S2a). Neurons were then further aged in vitro to day 47, but at this later timepoint the relative hyperexcitability of *C9* neurons vs CTRL neurons was not observed (Fig. 2ei,ii, Figure S2b). This loss of hyperexcitability phenotype appeared to be the result of an increase in AP firing frequency in the CTRL neurons from day 40 to day 47 to match the rate of the *C9* lines, rather than a decrease in the *C9* neuron excitability. Thus, the hyperexcitability phenotype observed at day 40 is transient, with the high AP frequency in *C9* lines reflecting a possible faster initial maturation rate in the *C9* lines than the CTRL lines.

### Increased excitability is not caused by altered voltage-gated currents

**Figure 3:**
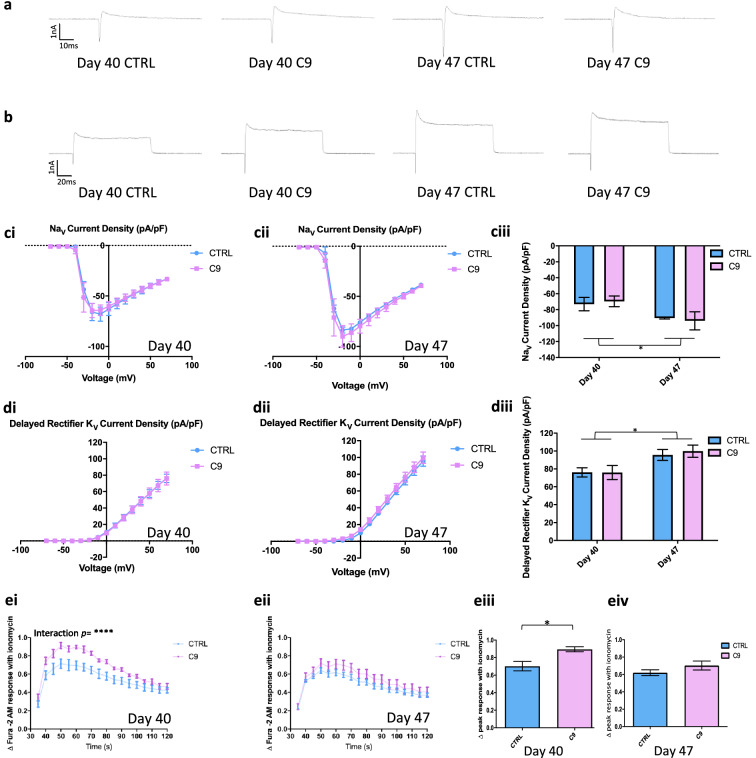
Transient calcium dyshomeostasis is evident in *C9ORF72* MNs vs CTRL MNs. Example traces for Na_V_ at − 20 mV (**a**) and K_V_ at + 50 mV. (**b**) Current densities show no significant difference between the CTRL or *C9* groups (Na_V_ IV graph, **ci**; K_V_ IV graph, **di**). The same measures also show no difference at day 47, (Na_V_ IV graph, **cii**; K_V_ IV graph, **dii**). However, a temporal comparison shows a significant increase of average peak current density of Na_V_ current and K_V_ current over time. Two-way RM ANOVA: Interaction Na^+^/K^+^ *p* = ns, Time *Na^+^ *p* = 0.0282, *K^+^ *p* = 0.0145, Genotype Na^+^/K^+^ *p* = ns with Sidak’s multiple comparisons Na^+^/K^+^ *p* = ns, N = 3 lines, 1–2 differentiations. Investigation of calcium store release (ei, eii; mean ± SEM) evoked by ionomycin injection showed a significant increase in the delta Fura-2 AM fluorescence of C9 versus CTRL at Day 40 (eiii; Two-way RM ANOVA: Interaction **** *p* = < 0.0001, Time *p* = < 0.0001, Genotype *p* = ns) but not Day 47 (eiv; Interaction *p* = ns, Time *p* = < 0.0001, Genotype *p* = ns.) Unpaired T-Test *p* * < 0.05, N = 3 lines, 2–3 differentiations.

We recorded fast inactivating Na^+^ and delayed rectifier K^+^ voltage gated-channel currents, which are involved in the upstroke and recovery of APs, respectively^21,22^. Na_V_ current and K_V_ current recordings were normalised to capacitance giving current densities and allowing us to assess whether functional expression of ion channels contributed to the hyperexcitability phenotype. At neither time point was there a significant difference in either Na^+^ or K^+^ current density between genotypes (Fig. 3a–d). However, a significant increase in peak current density was detected over time for both Na^+^ or K^+^ currents, suggesting a maturing population of neurons (Fig. 3ciii,diii). Overall, these results suggest that fast inactivating Na^+^ or delayed rectifier K^+^ channels do not play a significant role in the increased firing rate observed at day 40 in *C9*-MNs.

### Early hyperexcitability correlates with aberrant increased intracellular calcium release

Ca^2+^ release from internal neuronal stores supports multiple roles in the correct functioning of neurons and shapes neuronal output (e.g. AP frequency) through modulation of after-hyperpolarisations or after-depolarizations^23^.

We therefore investigated Ca^2+^ release from intracellular stores using the Fura-2AM ratiometric dye. When MNs were activated with ionomycin, we observed a typical biphasic Ca^2+^ response at both day 40 and 47 (Fig. 3ei,ii), due to initial release of Ca^2+^ from intracellular stores likely followed by store operated Ca^2+^ entry (SOCE)^24^. A significant change in delta Ca^2+^ release compared to baseline was detected in young neurons at day 40 (Fig. 3ei, Figure S3a). Specifically, the delta peak magnitude of Ca^2+^ release illustrated a significant increase in intracellular stored Ca^2+^ release from *C9*-MNs vs CTRL MNs at day 40 (Fig. 3eiii). Importantly, as with the electrophysiological phenotype observed at day 40, the difference in internal Ca^2+^ release was transient and no longer detected at day 47 (Fig. 3eii,iv, Figure S3b). Finally, we found a significant increase in Ca^2+^ release from the mitochondria in *C9*-MNs compared to CTRL MNs upon stimulation with FCCP. This difference in release properties was only observed at the later day 47 timepoint (Figure S4a-d) and may represent a later stage in the disease process involving the mitochondria.

## Discussion

This study focused on iPSC-derived MNs harbouring a *C9ORF72* expansion to assess electrophysiological excitability changes during neuronal maturation as a potential mechanism underlying the earliest stages of disease. We sought to provide clarity to a field in which several studies have used different parameters to define neuronal excitability and shown a mixture of hypoexcitability, hyperexcitability transitioning phenotypes, or observed no excitability changes at all^15–18^. We demonstrated increased evoked AP firing in *C9*-MNs at an early stage (day 40) of iPSC-derived MN maturation. This effect was, however, transient, with the induced AP firing frequency in the CTRL MNs increasing from day 40 to 47 resulting in equivalent AP frequency in both CTRL and *C9*-MNs at day 47. These changes in excitability may result from different rates of neurophysiological maturation, with *C9*-MNs reaching greater maturity at day 40 compared to CTRL MNs, a genotype-specific difference lost by day 47.

These data also provide a rationale for differing results in the field regarding *C9* neuronal excitability. The data here confirm that hyperexcitability exists as an early stage phenotype, as also suggested by Devlin et al.^17^. Since Wainger et al*.*^15^ and Devlin et al*.*^17^ observed hyperexcitability at a similar timepoint to us (at approximately day 38 of differentiation, or 2 weeks post re-plating, respectively) our hyperexcitability data at day 40 confirm the idea of an early disease-associated hyperexcitability phenotype. Devlin et al.^17^ noted hypoexcitability at 7–10 weeks post plating and Sareen et al*.*^16^ at 66–79 days of differentiation. That we observe the loss of a hyperexcitable phenotype in *C9*-MNs compared to CTRL MNs at day 47, illustrates a rapid change in excitability in only 7 days in vitro. This loss of phenotype could present an intermediate stage of disease progression between the hyper- and hypo-excitable states. Of note, the mutant *C9ORF72* iPSC lines used in this study differed from those used by Wainger et al*.*^15^*,* Sareen et al*.*^16^ and Devlin et al*.*^17^ in their studies, thereby demonstrating the hyperexcitability phenotype in young neurons is robust and not simply the result of studying a particular set of iPSC lines.

Both Na_V_ and K_V_ channels have been implicated in excitability changes in ALS patients and *C9ORF72* iPSC models^11,15–17,25,26^. However, in contrast, we found no change in fast inactivating Na_V_ or delayed rectifier K_V_ current. This does not rule out a contribution for Na^+^ or K^+^ current dysfunction as A-type K^+^ channels and the Na^+^/K^+^ pump could play a role. Our observations from day 40 to 47 show an increase in both Na_V_ and K_V_ current densities, indicating ongoing maturation of neuronal cultures in our study.

Ca^2+^ dyshomeostasis has strong links to ALS pathogenesis^18,19,27,28^. MNs are particularly vulnerable to Ca^2+^ dyshomeostasis due to high Ca^2+^ influxes during neurotransmission as well as their low overall Ca^2+^ buffering capability. Recent studies have shown AMPA receptors have increased Ca^2+^ permeability in *C9*-MNs which may contribute to excitotoxicity via increased [Ca^2+^]_i_ and subsequent activated cellular stress pathways^18,29^. Using Fura-2AM, Dafinca et al.^28^ showed iPSC-derived *C9* neurons to have increased internal Ca^2+^ release in response to ionomycin. In addition, the same study found that upon chemical neuronal depolarisation (KCl 50 mM), live cell imaging revealed increased Ca^2+^ release and delayed Ca^2+^ clearance from the cytosol in *C9* vs CTRL MNs. We show that the dyshomeostasis in internal Ca^2+^ release in response to ionomycin coincided with the presence of evoked hyperexcitability. One potential explanation may be that the effect of increased intracellular calcium during the period of neuronal maturation may impact expression of ion channel genes over time contributing to hyperexcitability.

Finally, in addition to perturbed Ca^2+^ release from intracellular organelles, we observed a change in stimulated mitochondrial Ca^2+^ release at day 47 in *C9-*MNs. Therefore, the loss of hyperexcitability at day 47 coincides with the loss of increased general intracellular organelle Ca^2+^ release and with the appearance of mitochondrial Ca^2+^ dysfunction. Mitochondria are important for ATP production as well as for Ca^2+^ homeostasis, free radical formation, metabolite generation and apoptosis^30^. Mitochondrial dysfunction has been previously implicated in ALS, particularly in SOD1 models^31^. However, a variety of aberrations, including reduced ATP production as well as disrupted architecture, are also observed in different ALS mutations as reviewed by Smith et al.^32^. Ca^2+^ overload is one mechanism proposed in ALS mitochondrial dysfunction and neuronal cell death pathway activation. Mitochondria rely on cross-talk with the ER for maintaining balanced Ca^2+^ concentration and Ca^2+^ signalling^31–33^. A shift in Ca^2+^ storage from the ER to mitochondria could lead to reduced Ca^2+^ in the ER, but increased Ca^2+^ in the mitochondria. Too little Ca^2+^ in the ER could result in protein misfolding, whereas too much Ca^2+^ in the mitochondria leads to opening of the ‘mitochondrial permeability transition pore’ and subsequent necrosis or apoptosis^34^. The shift from increased general intracellular organelle calcium release at day 40 to increased Ca^2+^ release specifically from the mitochondria at day 47 in *C9*-MNs compared to CTRL MNs could suggest the possibility of mitochondria Ca^2+^ overload later in the process of cellular degeneration.

The presence of astrocytes in iPSC-MN cultures and their impact on the biology of MNs carrying ALS mutations is an area of debate as highlighted by Zhao and colleagues^30^. In that paper the authors’ showed astrocytes are affected by the presence of the *C9ORF72* mutation which then contributes towards MN dysfunction. It should, therefore, be noted that the cultures used in our study include a mixed population of neurons and astrocytes as a result of the differentiation protocol used.

Overall, our study seeks to clarify the conflicting reports surrounding altered excitability in *C9ORF72* neurons. Here, we report transient disease-related hyperexcitability in a mixed ‘MN-Astrocyte culture’ which is directly affected by neuronal maturity. Our work associates the hyperexcitability phenotype to a temporally-linked dyshomeostasis in Ca^2+^ release, followed by a normalization of excitability and emergence of mitochondrial Ca^2+^ dysfunction at later stages. Together, these data provide a Ca^2+^ based neurophysiological phenotype which could be manipulated for therapeutic benefit in pre-symptomatic stages of disease.

## Methods

### iPSC culture and MN differentiation

iPSC lines were previously generated from patients carrying the *C9ORF72* expansion and control individuals^19^, then differentiated into MNs using the Du et al^20^ protocol with minor modifications. At day 28 the media was changed to basal media supplemented with 10 ng/ml IGF, 10 ng/ml CNTF, 10 ng/ml BDNF and 0.1 mM ascorbic acid.

### Immunostaining

Coverslips were fixed in 4% PFA, permeabilized with 0.1% triton (PBS-T) followed by 1 h blocking in PBS-T with 5% donkey serum and 2% bovine serum albumin. Primary antibodies (TUJ1-1:1000-Abcam; SMI-32-1:1000-Biolegend) were applied overnight at 4 °C. Secondary antibodies (Alexa 1:1000) were incubated for 1 h at room temperature. Cells were counterstained with 1 µg/ml DAPI then mounted in Vectashield.

### Flow cytometry

2.5 × 10^6^ iPSC-derived MNs were dissociated using Trypsin–EDTA and fixed in 4% PFA on ice for 10 min. Cells were blocked for 10 min in 1 M glycine and solution PB (0.1% Saponin, 2% BSA and Normal Donkey IgG in PBS). Blocked samples were split for staining. One sample used for the IgG control, one for unstained control and one for primary antibodies (Normal Mouse IgG; Normal Rabbit IgG; TUJ1; SMI-32). Antibodies were incubated for 1 h at 4 °C. Alexa secondary antibodies (1:2000) were incubated for 15 min on ice. Primary stained samples were compared against their IgG control for positive cells and analysed using FlowJo v10.4 Software (BD Life Sciences).

### RT-qPCR

1.25 × 10^6^ cells were used per RNA sample. Trizol reagent and chloroform were used along with the RNase Easy Mini Columns (Qiagen) with DNase I treatment. cDNA was produced using SuperScript VILO MasterMix, using 1 μg of RNA. Fast SYBR green was used for the qPCR reaction. Primer sequences in Supplementary Table 1 and data analysed using dCT method.

### Whole-cell patch clamp electrophysiology

10^5^ cells were plated per coverslip and selected for electrophysiology based on morphology. Neurons were selected for patch clamping both within and outside of neuronal clusters. Due to the enrichment of the culture for MNs the cells predominantly recorded were MNs. Although without live, cell-type specific labelling it cannot be excluded that some recordings may be from interneurons, the spread of passive properties data does not suggest a separation in cell types. Cells with R_a_ greater than 40 MΩ were rejected and recordings with a change in R_a_ or cell capacitance greater than 10% over the duration of the recording were omitted. Any cell lines post stained with less than 30% MNs were excluded.

Internal solution (mM): 140 Potassium Gluconate, 6 NaCl, 1 EGTA, 10 Hepes, 4 MgATP, 0.4 Na_3_GTP, adjusted to pH of 7.3 using KOH and osmolarity 290 mOsm. aCSF included (mM): 167 NaCl, 2.4 KCl, 1 MgCl_2_, 10 Glucose, 10 Hepes and 2 CaCl_2_ adjusted to a final pH of 7.4 and osmolarity of 300 mOsm.

Induced APs were recorded in current clamp by injecting 500 ms current steps in 5 pA increments from − 10 pA. Membrane potential was maintained around − 70 mV by injecting a hyperpolarizing bias current. Na_V_ and K_V_ currents were recorded in voltage clamp (sampling rate of 10,000 Hz) during a series of 10 mV voltage steps, 100 ms in duration, from a holding potential of − 70 mV. Leak subtraction was applied with 4 sub-sweeps and a settling time of 250 ms.

### Organelle calcium release

Neurons were plated at a density of 10^5^ cells per well on 96-well plates. Stimulation by ionomycin and recordings using Fura-2 AM were performed as described by Wallings et al.^35^.

### Analysis

For the above techniques, data from multiple lines and differentiations have been pooled to allow more robust analysis with a higher number of cells and greater statistical power. Patch clamp electrophysiology is a low-throughput technique, and a much higher number of differentiations and patched neurons would be required to present separate data for individual lines. Key figures can be found in the supplementary data, displayed with each line separately for data transparency. Authors can confirm that all relevant data are included in the paper and/ or its supplementary information files.

## Supplementary Information

Supplementary Information 1.

Supplementary Information 2.
