# Supplementary material for: Hyperexcitability in young iPSC-derived C9ORF72 mutant motor neurons is associated with increased intracellular calcium release
Source: Sci Rep. 2022 May 5;12:7378. doi: 10.1038/s41598-022-09751-3 (PMC9072315; doi:10.1038/s41598-022-09751-3)
Supplement: Supplementary file 1 — Supplementary Information 1. [file 41598_2022_9751_MOESM1_ESM.pdf]

Figure S1 Burley et al

a

Label	Identification Code	Method of Reprogramming	Age	Gender	Genotype
CTRL 1	856-03-04	Cytotune (SeV)	78	F	Control
CTRL 2	AH017-3	Sendai virus (SeV)	67	F	Control
CTRL 3	065-03-03	Cytotune 2 (SeV)	65	M	Control
C9-1	OXC9-04-01	Cytotune (SeV)	39	M	ALS
C9-2	OXC9-01-06	Cytotune (SeV)	72	M	ALS-FTD
C9-3	OXC9-02-02	Cytotune (SeV)	62	F	ALS-FTD

b

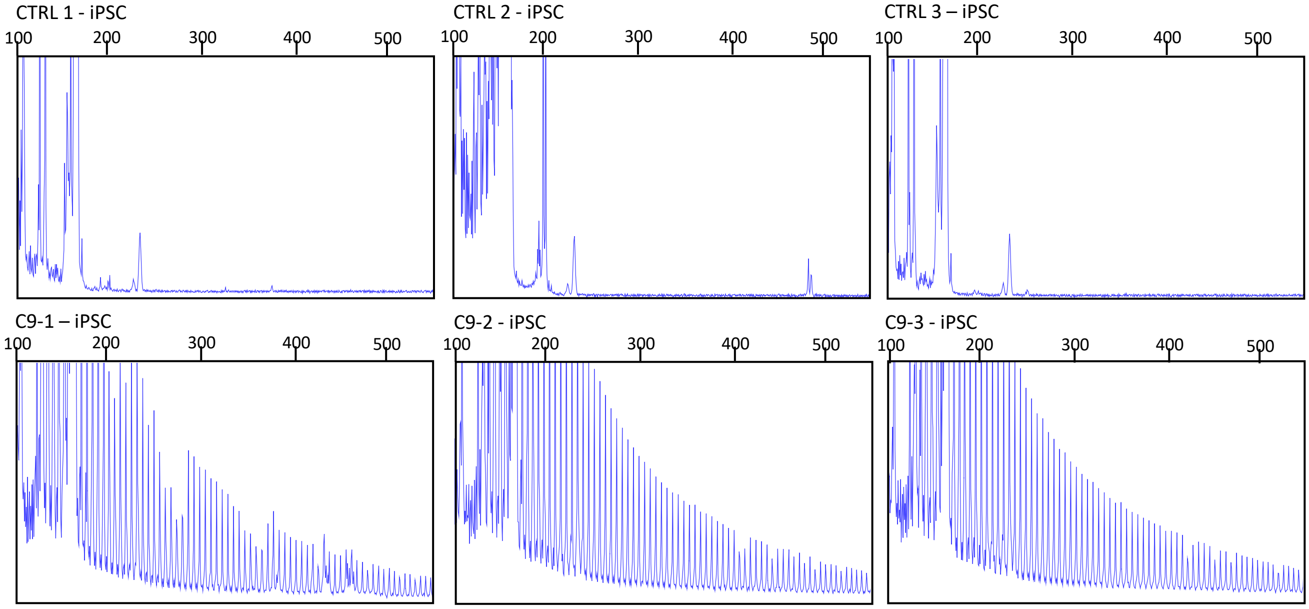

c

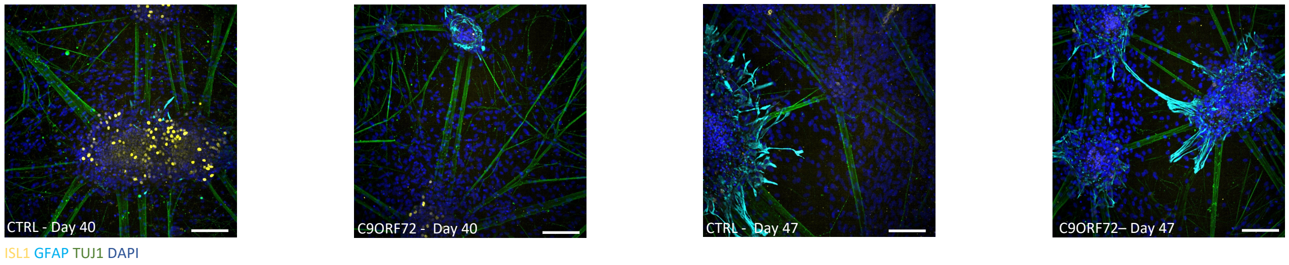

Figure S2

Burley et al

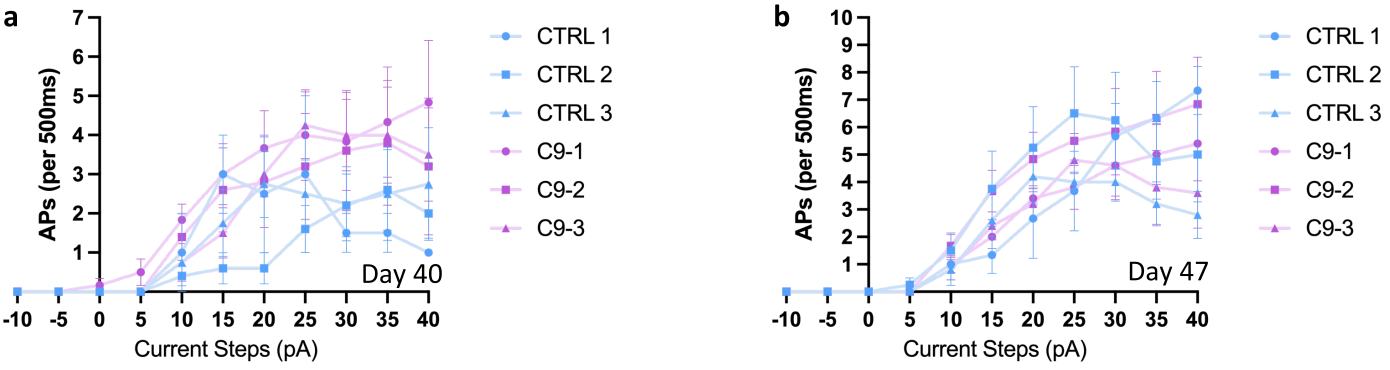

Figure S3

Burley et al

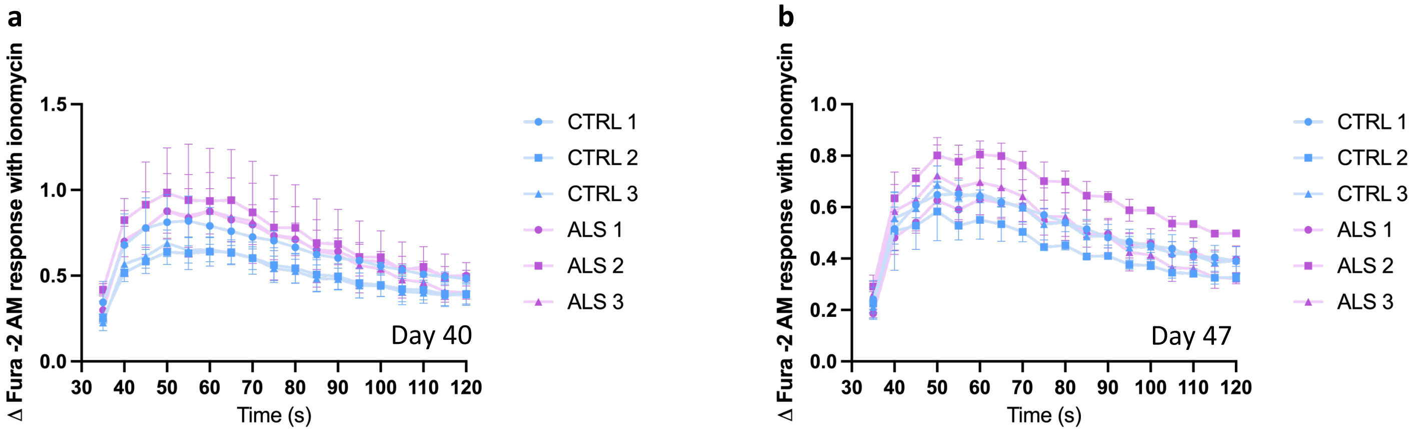

Figure S4

Burley et al

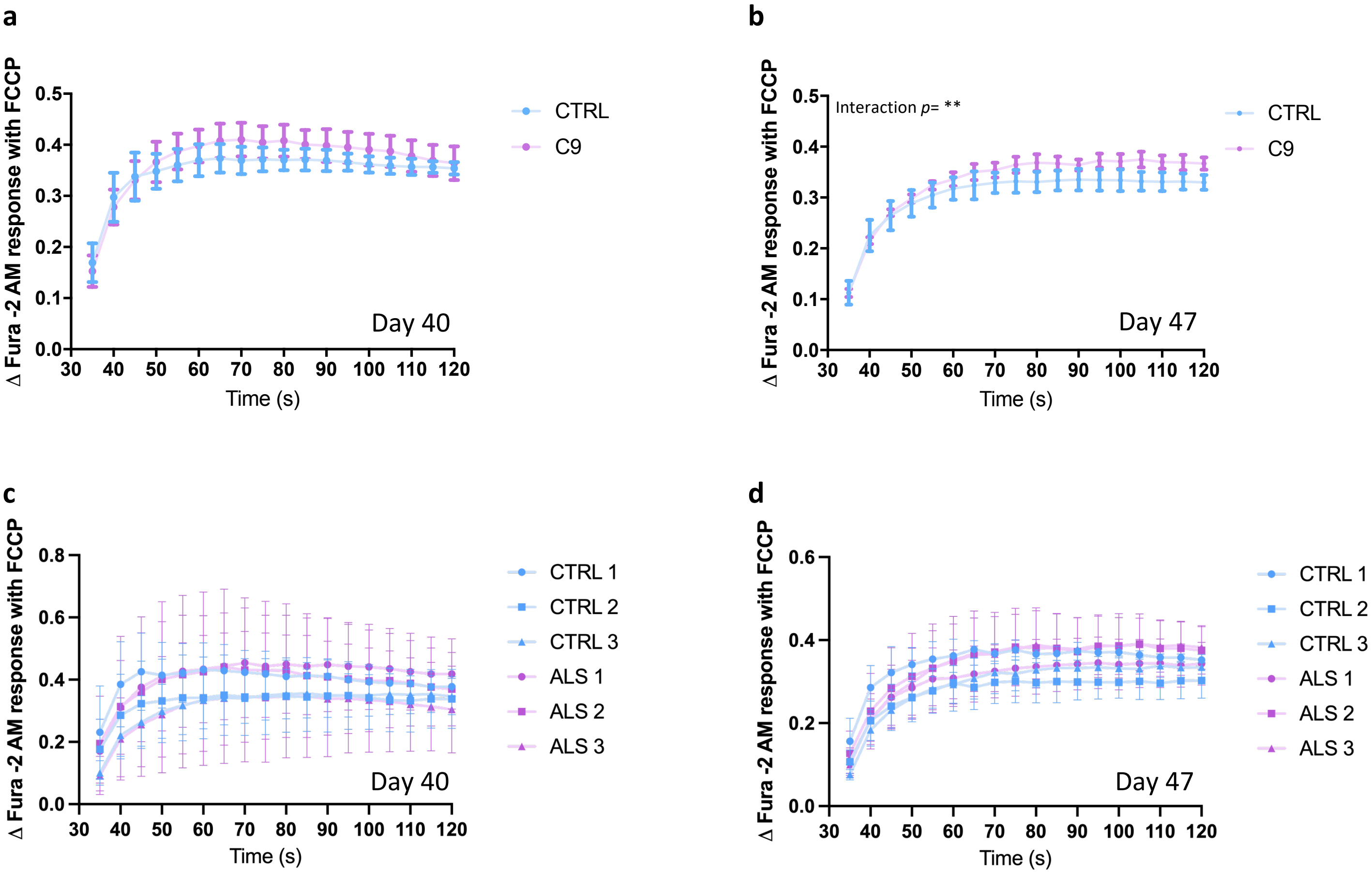

Gene	Sequence
SMI-32	F: ACCGTCATCAGGCCGACATT<br>R: ATCTCCCACTTGGTGTTCCTC
ISL1	F: TCAGTATTTGGACGAGAGCTGTA<br>R: CGTTCTTGCTGAAGCCGATG
HB9	F: GCTCATGCTCACCGAGACCC<br>R: TCTTTGGCCTTTTTGCTGCGT
HPRT	F: GCTATAAATTCTTTGCTGACCTGCTG<br>R: AATTACTTTTATGTCCCCTGT TGACTGG
