# Supplementary material for: Hyperexcitability in young iPSC-derived C9ORF72 mutant motor neurons is associated with increased intracellular calcium release
Source: Sci Rep. 2022 May 5;12:7378. doi: 10.1038/s41598-022-09751-3 (PMC9072315; doi:10.1038/s41598-022-09751-3)
Supplement: Supplementary file 2 — Supplementary Information 2. [file 41598_2022_9751_MOESM2_ESM.pdf]

Hyperexcitability in young iPSC-derived *C9ORF72* mutant motor neurons is associated with increased intracellular calcium release

Sarah Burley[1,2], Dayne A Beccano-Kelly[1,3], Kevin Talbot[4], Oscar Cordero Llana[1,5], and Richard Wade-Martins[1*]

[1] Department of Physiology, Anatomy and Genetics, University of Oxford, South Parks Road, Oxford, UK

[2] Current address: School of Biology, University of St Andrews, North Haugh, St Andrews, UK

[3] Current address: UK Dementia Research Institute, University of Cardiff, Hadyn Ellis Building, Maindy Road, Cardiff, CF24 4HQ

[4] Nuffield Department of Clinical Neurosciences, University of Oxford, John Radcliffe Hospital, Oxford, UK

[5] Current address: Bristol Medical School, Translational Health Sciences, University of Bristol, Dorothy Hodgkin Building, Whitson Street, Bristol BS1 3NY

* To whom correspondence should be addressed: richard.wade-martins@dpag.ox.ac.uk

Supplementary Information

Supplementary Methods

Repeat Primed PCR

Repeat Primed PCR is used to report the presence or absence of the hexanucleotide repeat expansion in the *C9ORF72* gene. DNA was extracted for repeat primed PCR using Phenol: Chloroform extraction. Cells were lysed in lysis buffer containing: 0.6% SDS, 100 mM NaCl, 50 mM TrisCl (pH 8) and 20 mM EDTA with 50 mg/ml RNase A for 20 minutes RT, then left overnight at 37 °C. Proteinase K treatment followed at 100 µg/ml O/N at 37 °C. Lysate was extracted 2x with Phenol: Chloroform: Isoamyl Alcohol and 2x with Chloroform using Light Phase Lock Gel tubes (Eppendorf). DNA was then precipitated with 5 M NaCl and absolute Ethanol and centrifuged at 17950 g at 4 °C for 30 minutes. The pellet was washed with 70% ethanol and spun for 30 minutes at 4 °C. After removing the 70% ethanol and drying the pellet the DNA was resuspended in 75 ml TE for repeat primed PCR. The repeat primed PCR cycling conditions and primers followed the DeJesus-Hernandez *et al.*[1] and Hantash *et al.*[2] method followed by fragment analysis using the Liz-500 ladder. Results of fragment analysis were analyzed using Geneious.

Supplementary information references

1. DeJesus-Hernandez M, Mackenzie IR, Boeve BF, et al. Expanded GGGGCC Hexanucleotide Repeat in Noncoding Region of C9ORF72 Causes Chromosome 9p-Linked FTD and ALS. *Neuron*. 2011;72(2):245-256. doi:10.1016/J.NEURON.2011.09.011

2. Hantash FM, Goos DG, Tsao D, et al. Qualitative assessment of FMR1 (CGG)n triplet repeat status in normal, intermediate, premutation, full mutation, and mosaic carriers in both sexes: Implications for fragile X syndrome carrier and newborn screening. *Genet Med*. 2010;12(3):162-173. doi:10.1097/GIM.0b013e3181d0d40e

Supplementary Figure Legends

Figure S1: StemBancc *C9ORF72* iPSC and CTRL line characteristics

(a) Donor information from the three C9 and three CTRL iPSC lines selected for the study including age of donor when sample was taken, gender, method to reprogram cells and clinical disease classification. F = Female, M = Male, SeV = Sendai virus. (b) The presence of the *C9ORF72* gene repeat expansion in patient iPSCs and lack of expansion in control iPSCs was confirmed using repeat primed PCR. Y-axis inset increased to 2000 relative fluorescent units. X-axis in base pairs. (c) iPSC-derived MNs immunostained for GFAP and ISL1. Scale bars = 100 μm.

Figure S2: Hyperexcitability is present in young *C9ORF72* MNs vs CTRL MNs

(a, b) Average action potential firing per 500 ms for each current step (mean ± SEM). Data presented with iPSC lines separated for visualization purposes only, statistics of pooled data shown in main figure.

Figure S3: Internal calcium release in response to Ionomycin

Fura-2 AM calcium assay with Ionomycin injected at 30 seconds and baseline corrected (mean ± SEM). (a, b) Data presented with iPSC lines separated for visualization purposes only, statistics of pooled data shown in main figure.

Figure S4: Internal calcium release in response to FCCP

Fura-2 AM calcium assay with FCCP injected at 30 seconds and baseline corrected (mean ± SEM). (a, b) Data Two-way RM ANOVA: Day 40: Interaction p= ns, Time p= <0.0001, Genotype p= ns, Day 47: Interaction p= 0.0072, Time p= <0.0001, Genotype p= ns, N= 3 lines, 3 differentiations. (c, d) Data presented with iPSC lines separated for visualization purposes only, statistics of pooled data shown in main figure.

Table S1: Primers used for qPCR
